# Does Survival Vary for Breast Cancer Patients in the United States? A Study from Six Randomly Selected States

**DOI:** 10.1155/2017/6950579

**Published:** 2017-07-26

**Authors:** Hafiz M. R. Khan, Lisaann S. Gittner, Abhilash Perisetti, Anshul Saxena, Aamrin Rafiq, Kemesha Gabbidon, Sarah Mende, Maria Lyuksyutova

**Affiliations:** ^1^Department of Public Health, Texas Tech University Health Sciences Center, Lubbock, TX 79430, USA; ^2^Department of Political Science, Texas Tech University, Lubbock, TX 70409, USA; ^3^Department of Family and Community Medicine, Texas Tech University Health Sciences Center, Lubbock, TX 79430, USA; ^4^Department of Health Promotion and Disease Prevention, Florida International University, Miami, FL 33199, USA; ^5^Department of Computer Science, Texas Tech University, Lubbock, TX 79409, USA; ^6^School of Medicine, Texas Tech University Health Sciences Center, Lubbock, TX 79430, USA

## Abstract

**Background:**

Breast cancer is the most common cancer in women. Disparities in some characteristics of breast cancer patients and their survival data for six randomly selected states in the US were examined.

**Materials and Methods:**

A probability random sampling method was used to select the records of 2,000 patients from each of six randomly selected states. Demographic and disease characteristics were extracted from the Surveillance Epidemiology and End Results (SEER) database. To evaluate relationships between variables, we employed a Cox Proportional Regression to compare survival times in the different states.

**Results:**

Iowa had the highest mean age of diagnosis at 64.14 years (SE = 0.324) and Georgia had the lowest at 57.97 years (SE = 0.313). New Mexico had the longest mean survival time of 189.09 months (SE = 20.414) and Hawaii the shortest at 119.01 (SE = 5.394) months, a 70.08-month difference (5.84 years). Analysis of stage of diagnosis showed that the highest survival times for Whites and American Indians/Alaska Natives were for stage I cancers. The highest survival times for Blacks varied. Stage IV cancer consistently showed the lowest survival times.

**Conclusions:**

Differences in breast cancer characteristics across states highlight the need to understand differences between the states that result in variances in breast cancer survival.

## 1. Introduction 

 Breast cancer is a worldwide public health concern within many countries, including the United States (US) which has experienced a recent 20% increase in breast cancer diagnosis. Breast cancer is the most prevalent type of cancer (29%) in women in the US. Additionally, breast cancer is the most commonly diagnosed malignancy among women in the US, accounting for nearly one out of every three diagnosed malignancies [1, 2]. Between 1975 and 1995, breast cancer incidence in women increased from 81 out of 100,000 person-years to 97 out of 100,000 person-years (Althuis, Dozier, Anderson, Devesa, and Brinton, 2005). Secondly, the US had a 29% rate of breast cancer in women, with women over the age of 50 years having the highest incidence ([3]; Kohler et al., 2015). Furthermore, it is estimated that one in eight women living in the US will develop breast cancer in their lifetime [1]. In 2013, there were 232,340 cases of invasive breast cancer in women in the US and 39,620 associated deaths [1]. In the US, over three million women are currently living with a history of invasive breast cancer, with 40% of cases occurring in women over 65 years of age and 20% among women younger than 50 years of age [4]. Therefore, the increase in breast cancer incidence and the cancer-related complications have remained a significant public health issue within the US over the last few decades.

*Prevention and Treatment*. Early detection is the primary focus of breast cancer prevention efforts in most settings. In 2015, the American Cancer Society (ACS) reported that 66% of women aged 40 years and older had a mammogram within the past two years [5], an increase from 2010 where it was estimated that at least half of all women in the US between ages 40 and 74 years had received a mammogram [6].

The National Breast and Cervical Cancer Early Detection Program (NBCCEDP) is a program developed by the Centers for Disease Control and Prevention (CDC) to increase breast and cervical cancer screening rates among economically disadvantaged women. In the years 2011 to 2012, NBCCEDP screened 549,043 underserved women between the ages of 40 and 64 years. NBCCEDP screened about 10% of eligible women with screening rates ranging from 3.2 to 52.8% of the eligible population [7]. Overall, the program screened over half a million women; however, most eligible women remained unscreened for breast cancer and may require increased outreach efforts [7]. Mammogram use was lowest among American Indians and Alaska Natives at 36% [7, 8]. Research shows that 43.5% of the 137,274 eligible women had at least one mammogram screening. Additionally, women 66 to 74 years old were more likely to get mammography screening compared to those 85 to 100 years or older (57.2% versus 15.2%, resp.; *p* < 0.001) [9]. Further research shows that 50.1% of Black women and 40.8% of White women aged 65–74 years received either no or one screening mammogram from 2005 to 2008 [10].

*Health Disparities*. There have been major improvements in screening and treatment; however, there remain racial and ethnic differences in breast cancer screening and mortality. As of 2014, the rates of breast cancer have increased for Black Americans and have decreased for Hispanics [1]. The public health community has focused on increasing the breast cancer screening rates of ethnic and racial minorities and has had some success increasing the rates of breast cancer screening for Black Americans and some Hispanic groups, with the exception of Mexican Americans [11]. Ethnicity and race are found to be major predictors of breast cancer prognosis and incidence; social, environmental, and hereditary determinants directly affect the development of breast cancer [3, 6, 8, 12, 13].

Black women have an increased risk for more aggressive forms of breast cancer, such as estrogen receptor negative tumors, which frequently do not respond well to current therapies [6]. Black women under the age of 50 years also experience higher rates of breast cancer compared to White women of comparable ages. Additionally, Black and White women who had one mammogram annually had a lower 10-year mortality than those who received screening irregularly or biennially [10]. When diagnosed at the same stage, Black women face higher rates of mortality associated with breast cancer than White women and are more likely to be diagnosed at advanced stages of the disease [14]. Mammogram use is 33% lower among immigrants who migrated to the US within the last 10 years [8]. Many of these immigrants lack health insurance and have lower education levels and limited income, which can contribute to lower screening rates, and women who experience inconsistent screening rates have a shorter 10-year survival time after breast cancer diagnosis [8, 11].

Structural, organizational, and political factors further exacerbate issues of racial and ethnic disparities in cancer mortality [14]. Issues of poverty, poor access to care, poor transportation, low or no income, and lack of health insurance increase the chances of a poor prognosis and lower screening rates [14]. Additionally, some groups are diagnosed with varying types of breast cancer. For example, estrogen receptor negative breast cancers have decreased across all ethnicities, but rates of estrogen receptor positive breast cancer have increased in young White women, older Hispanic women between the ages of 60 and 69 years, and all groups of Black Americans except the eldest groups [14, 15].

Some variations among populations of breast cancer patients are a result of state-level policy or environmental risks; because of these differences it is important to know what differences are present from state to state. This study uses cancer registries from six randomly selected states to perform statistical analyses. The registries are from Georgia, New Mexico, Hawaii, Connecticut, Iowa, and California. Between years 1999 and 2011, New Mexico and Iowa had an interval of 107.2 to 118.3 breast cancer cases per 100,000 people; Hawaii and Connecticut had between 126.9 and 141.4 cases per 100,000; and Georgia and California had 126.9 to 141.4 cases per 100,000 people. Death rates in each state ranged from 15.5 to 22.6 per 100,000. Hawaii, Connecticut, and New Mexico have death rates of 15.5 to 19.1 per 100,000; Iowa and California have rates from 19.2 to 21.1 per 100,000; and finally, Georgia have rates of 21.2 and 22.6 per 100,000 breast cancer cases [16]. In this paper, we provide the observed numbers of breast cancer cases and deaths in the US from 1973 to 2011 for six states randomly selected out of nine recorded states, as well as a broad summary of breast cancer incidence and survival times.

## 2. Materials and Methods

The study used data from the Surveillance Epidemiology and End Results (SEER) database (1973–2011). The SEER database started collecting data in 1973 for about 10% of the US population from nine states. Currently, the SEER program collects and publishes cancer incidence and survival information from cancer registries covering 28% of the US population [17]. The SEER website includes data from twenty population-based registries across varying states and territories. For this study, only data collected from the years 1973 to 2011 will be evaluated from each of the six states: California, Connecticut, Georgia, Hawaii, Iowa, and New Mexico. The representative probability sample data were randomly selected from the six SEER registries, and the selected data were summarized for information on stage of cancer, overall survival, and the lifetime probability of developing breast cancer.

Inclusion criteria for the present study are female gender, first and primary diagnosis of stages I, II, or III breast cancer, no previous cancer(s) being registered, and age 20 years or older. A participant's contribution to the person-years at risk began from the date of breast cancer diagnosis to the date of death or loss to follow-up, whichever occurred first. Women diagnosed with breast cancer at autopsy were excluded. Since breast cancer is uncommon in males, only female cases were included in this study. The SEER-coded categories of registry ID (REG) were used to classify participants into six mutually exclusive categories. Simple random sampling was used to select 2,000 cases from each registry. Information on the methods used for random sampling can be found in previously published literature by Khan et al. [18–21]. In addition, we used subject demographic information (age at diagnosis, marital status, race, and ethnicity) and survival time from the SEER dataset for statistical analysis. Data regarding other socioeconomic factors, such as income and health insurance status, were not available.

**Figure 1:**
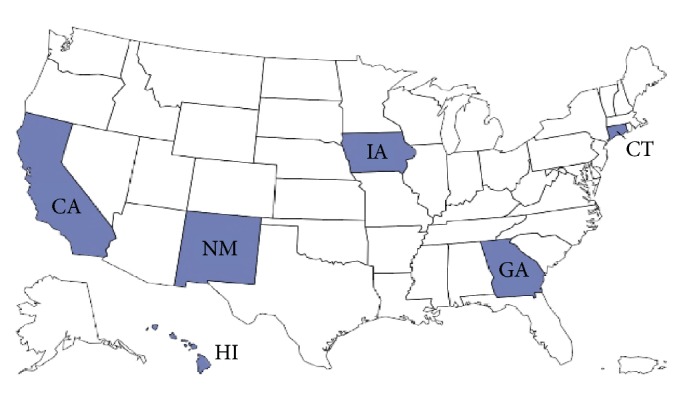
Female breast cancer cases (*n* = 2,000) randomly selected from the six states (darker blue color represents the selected states).

A total of 12,000 women with breast cancer were included in the analysis (for each state's registry *n* = 2,000, Figure 1) and individual survival time is defined by** t**, where** t** contains 12,000 survival data points and *n* = 2,000 for each state. Survival analysis accounted for both censored (patients who survived till the end of SEER registry's cutoff date) and uncensored (any patient who died within the SEER registry's cutoff date) data. Survival time was calculated in months using the Cox proportional hazards model, adjusting for age at diagnosis, race, ethnicity, and marital status. Cox proportional hazards models generated the adjusted hazard ratio and their 95% confidence intervals (CI). Data analysis was conducted using SPSS software (IBM SPSS for Windows version 20, 2011) and SAS® software version 9.4.

## 3. Results and Discussion

Using a probability sampling method, which is simple random sampling method, 2,000 patients were selected from six state cancer registries (California, Connecticut, Georgia, Hawaii, Iowa, and New Mexico). Tables 1 and 2 contain the descriptive statistics.  Table 1 shows diagnosis (in years), survival time (in months), and marital status at the time of diagnosis. Iowa has the highest mean age of diagnosis, 64.14 years (SE = 0.324), and then Connecticut, California, New Mexico, Hawaii, and Georgia, with mean age of diagnosis being 62.51 (SE = 0.320), 61.42 (SE = 0.564), 61.3 (SE = 0.316), 59.72 (SE = 0.305), and 57.97 (SE = 0.313), respectively.  Table 1 also indicates that Iowa has the older stage at diagnosis for breast cancer patients followed by Connecticut. Hawaii and Georgia have the lowest ages of breast cancer diagnosis. The 25th and 50th quartiles of age at diagnosis ranged from 48–53 to 57–65 years of age, respectively, for all six states.

Mean survival days (in months) were also calculated and stratified by each cancer registry. New Mexico reported the longest mean survival time of 189.09 months (SE = 20.414), and Hawaii reported the shortest mean survival time of 119.01 (SE = 5.394) months, representing a 70.08-month difference (5.84 years). The 25th and 50th quartiles of survival times generally ranged from 33–41 to 83–96 months, respectively, for all six states.

Table 2 shows the frequency and percentage of each race, ethnicity, and marital status for each of the six states studied. Most participants were married with widowed being the second most common relationship status, and separated women were the smallest group. Married women made up over half of the women with a breast cancer diagnosis, ranging from 52.95% in Connecticut to 59.6% in Hawaii.

We have stratified data by race and 5-year-time intervals for age at diagnosis and obtained the following summary statistics for six states: in California during 1973–1980, 61.50 years (SD = 13.68) was the mean age of diagnosis for White patients, compared to 57.52 years (SD = 14.47) for Black patients and 47.71 years (SD = 18.09) for American Indians/Alaska Natives. Between 1981 and 1985, American Indians/Alaska Natives had a mean age of diagnosis of 54.18 years (SD = 13.34) for diagnosis compared to 52.45 years (SD = 15.51) for Blacks and 63.42 years (SD = 14.34) for Whites. During 1986–1990, Whites had a mean age of diagnosis of 61.57 years (SD = 15.02) as their mean age of diagnosis compared to 55.44 years (SD = 18.14) for Blacks and 53.42 years (SD = 14.99) for American Indians/Alaska Natives. During 1991–1995, American Indians/Alaska Natives had a mean age of diagnosis of 55.56 years (SD = 15.25), compared to 59.76 years (SD = 14.21) for Blacks and 62.19 years (SD = 13.86) for Whites. During 1996–2000, American Indians/Alaska Natives had a mean age of diagnosis at 62.31 years (SD = 15.39) compared to 55.78 years (SD = 11.70) for Blacks and 61.54 years (SD = 12.65) for Whites. During 2001–2005, Whites had a mean age of diagnosis at 60.45 years (SD = 13.79), compared to 55.21 years (SD = 14.52) for Blacks and 57.43 years (SD = 12.77) for American Indians/Alaska Natives. During 2006–2011, Whites had a mean age of diagnosis at 62.47 years (SD = 13.93), compared to 64.53 years (SD = 11.99) for Blacks and 58.54 years (SD = 13.44) for American Indians/Alaska Natives.

In Connecticut between 1973 and 1980, Whites had 61.56 years (SD = 12.42) as the mean age of diagnosis compared to 47.88 years (SD = 16.83) for Blacks while no cases were reported for American Indians/Alaska Natives. During 1981–1985, American Indians/Alaska Natives had a mean age of diagnosis of 59 years with only one patient compared to 61.50 years (SD = 12.72) for Blacks and 62.86 years (SD = 14.79) for Whites. During 1986–1990, Whites had a mean age of 63.39 years (SD = 14.29) compared to 58.92 years (SD = 14.94) for Blacks and 61 years for American Indians/Alaska Natives with only one patient reported. During 1991–1995, there were no cases reported for American Indians/Alaska Natives, while the mean age for Blacks was 53.60 years (SD = 15.33) compared to 63.15 years (SD = 14.81) for Whites. Between 1996 and 2000, Whites had a mean age of diagnosis at 63.46 years (SD = 14.73) compared to 55.47 years (SD = 14.62) for Blacks and 76.00 years for American Indians/Alaska Natives with only one patient. During 2001–2005, there were no cases reported for American Indians/Alaska Natives, while the age of diagnosis for Blacks was 63.74 years (SD = 15.17) compared to 62.93 years (SD = 14.38) for Whites. During 2006–2011, Whites had a mean age of diagnosis at 61.90 years (SD = 14.13) compared to Blacks at 61.81 years (SD = 15.14) and American Indians/Alaska Natives at 59.17 years (SD = 14.63).

In Hawaii between 1973 and 1980, White patients reported 54.69 years (SD = 13.10) as the mean age of diagnosis compared to 64.00 years (SD = 1.414) for Blacks and 53.54 years (SD = 13.46) for American Indians/Alaska Natives. During 1981–1985, American Indians/Alaska Natives reported 54.85 years (SD = 12.47) for their age of diagnosis compared to 58.00 years (SD = 16.971) for Blacks and 58.56 years (SD = 15.47) for Whites. During 1986–1990, Whites had a mean age of diagnosis at 58.70 years (SD = 15.06) compared to 42.00 years (SD = 7.07) for Blacks and 59.25 years (SD = 12.82) for American Indian/Alaska Natives. During 1991–1995, American Indians/Alaska Natives had a mean age of diagnosis at 58.09 years (SD = 13.07) compared to 43.00 years for Blacks with only one patient reported and 61.30 years (SD = 13.65) for White patients. During 1996–2000, Whites reported 62.07 years (SD = 13.96) as their mean age of diagnosis compared to 53.00 years for Blacks with only one patient reported and 60.30 years (SD = 13.66) for American Indians/Alaska Natives. Between 2001 and 2005, American Indians/Alaska Natives reported 61.57 years (SD = 14.14) as the mean age of diagnosis compared to 51 years for Blacks with one patient reported and 62.11 years (SD = 14.13) for Whites. During 2006–2011, Whites reported 61.10 years (SD = 11.67) compared to 46.38 years (SD = 9.84) for Blacks and 61.09 years (SD = 13.06) for American Indians/Alaska Natives as the mean age of diagnosis.

In Iowa, during 1973–1980, Whites reported age 64.64 years (SD = 14.45) as mean age of diagnosis compared to 68.50 years (SD = 26.16) for Blacks while no cases were reported for American Indians/Alaska Natives. Between 1981 and 1985, no cases were reported for American Indians/Alaska Natives and Blacks while Whites' mean age of diagnosis was 64.24 years (SD = 14.26). During 1986–1990, Whites had a mean age of diagnosis of 65.83 years (SD = 14.46) compared to age 64 years for Blacks with one patient reported and no patients reported for American Indians/Alaska Natives. During 1991–1995, no cases were reported for American Indians/Alaska Natives while age 52.67 years (SD = 26.08) was reported for Blacks and 63.32 years (SD = 14.58) for Whites. During 1996–2000, Whites had a mean age of diagnosis at 64.70 years (SD = 14.96), while no cases were reported for Blacks during that time or American Indians/Alaska Natives. During 2001–2005, there were also no cases reported for American Indians/Alaska Natives; Blacks reported a mean of diagnosis at age 56.33 years (SD = 8.35) compared to Whites at age 64.30 years (SD = 14.64). Between 2006 and 2011, Whites had a mean age of diagnosis at 63.29 years (SD = 13.88) compared to 50.17 years (SD = 13.73) for Blacks and age 66 years among American Indians/Alaska Natives with only one patient reported.

In New Mexico, during 1973–1980, Whites had a mean age of diagnosis at 60.65 years (SD = 14.29) compared to 53.25 years (SD = 5.31) for Blacks and 57.50 years (SD = 17.05) for American Indians/Alaska Natives. During 1981–1985, American Indians/Alaska Natives had a mean age of diagnosis at 48.33 years (SD = 20.20) compared to 51 years for Blacks with only one patient reported and 60.57 years (SD = 14.98) for Whites. During 1986–1990, Whites reported a mean age of diagnosis at 62.23 years (SD = 14.50); Blacks reported no cases at that time and American Indians/Alaska Natives reported 53.60 years (SD = 16.33) as the age of diagnosis. During 1991–1995, American Indians/Alaska Natives reported age 65 years (SD = 13.10) compared to 80 years (SD = 7.07) for Blacks and 59.98 years (SD = 14.93) for Whites. During 1996–2000, Whites reported 61.28 years (SD = 13.93) as the mean age of diagnosis compared to 47.67 years (SD = 8.08) for Blacks and 60.88 years (SD = 9.52) for American Indian/Alaska Natives. During 2001–2005, American Indians/Alaska Natives had a mean of 55.59 years (SD = 11.03) compared to Blacks at 61.67 years (SD = 19.55) and Whites at 62.07 years (SD = 14.16). Between 2006 and 2011, Whites reported a mean age of diagnosis at 62.66 years (SD = 13.27) compared to Blacks at 44 years (SD = 15.53) and American Indians/Alaska Natives at 55.15 years (SD = 11.88).

In Georgia, during 1973–1980 Whites had a mean age of diagnosis at 58.67 years (SD = 13.84) compared to Blacks at 53.55 years (SD = 15.94) and American Indians/Alaska Natives at 53 years but with only one patient reported. During 1981–1985, no cases of American Indians/Alaska Natives were reported for that period; Blacks had a mean age of diagnosis at 55.20 years (SD = 17.89) compared to Whites at 59.39 years (SD = 15.76). During 1986–1990, Whites had a mean of 59.34 years (SD = 15.04) compared to Blacks at 52.78 years (SD = 13.84) while no cases were reported for American Indians/Alaska Natives for that period. During 1991–1995 American Indians/Alaska Natives had a mean age of 48.50 years (SD = 0.707) compared to 53.95 years (SD = 15.05) for Blacks and 59.64 years (SD = 13.75) for Whites. During 1996–2000, Whites reported a mean age of diagnosis at 59.49 years (SD = 12.73) compared to Blacks at 55.62 years (SD = 15.21) and 55.25 years (SD = 9.91) for American Indians/Alaska Natives. During 2001–2005, American Indians/Alaska Natives had 44.00 years (SD = 11.93) as the mean age of diagnosis compared to Blacks at 52.67 years (SD = 13.44) and Whites at 59.92 years (SD = 13.51). During 2006–2011, Whites had a mean of 59.44 years (SD = 13.57) for diagnosis compared to 56.37 years (SD = 12.68) and 49.63 years (SD = 11.56) for American Indians/Alaska Natives.

Data stratified by race and 5 years' period for age at diagnosis gave evidence of change of mean age of diagnosis across the span of 38 years. In New Mexico during 1973–1980 the mean age of diagnosis for Black patients was 53.25 years (SD = 5.31), while during 2006–2011 Black patients reported a mean age of diagnosis of 44 years (SD = 15.53). The White patients did not have such a difference reporting a mean age of diagnosis of 60.65 (SD = 14.29) during 1973–1980 and 62.66 (SD = 13.27) during 2006–2011. Hawaii additionally had similar results reporting mean age of diagnosis of 64.00 (SD = 1.414) for Black patients during 1973–1980 and 46.38 years (SD = 9.84) for Black patients during 2006–2011. White patients reported 54.69 years (SD = 13.10) as their mean age of diagnosis during 1973–1980 and 61.10 years (SD = 11.67) during 2006–2011. Stage 1 cancer at diagnosis had the longest survival for Whites and American Indians/Alaska Natives in 1986–1990, while Blacks had the longest survival for diagnosis at stage IIB. In 2001–2005, Stage 1 cancer at diagnosis had the longest survival for Whites and American Indians/Alaska Natives, but stage IIIA cancer had the longest survival for Blacks. Stage IV cancers had the shortest survival times across all ethnic groups between 1986–1900 and 2001–2005.

Table 3 shows the analysis of Cox proportional hazard ratio and its corresponding confidence intervals. New Mexico, with the highest mean survival time, was made the referent group for calculating hazard ratios, adjusting for states using the Cox Proportional Regression. Hazard ratios compare the probability of an event occurring in one group versus another group considering the time elapsed until the event occurs. Hazard ratios were calculated for five states. The hazard ratio for California is 0.925 with a 95% confidence interval (0.842, 1.017), with a *p* = 0.105. The hazard ratio for Connecticut is 0.991 with a 95% confidence interval (0.903, 1.087), with a *p* = 0.844. The hazard ratio for Georgia is 0.988 with a 95% confidence interval (0.895, 1.091), with a *p* = 0.811. The hazard ratio for Hawaii is 0.786 with a 95% confidence interval (0.711, 0.869), with a *p* < 0.0001. The hazard ratio for Iowa is 0.968 with a 95% confidence interval (0.884, 1.060), with a *p* = 0.484. In survival analysis, the event under consideration is death, and “alive” status was used as the censoring variable. The mean survival time for Hawaii was 119.01 months (9.92 years). New Mexico had a longer survival time of 189.09 months (15.75 years) when compared to the other states. Breast cancer patients who resided in California, Connecticut, Georgia, Hawaii, Iowa, and New Mexico had mean survival times of 181.12 months, 165.18 months, 155.13 months, 119.01 months, 152.76 months, and 189.09 months, respectively. New Mexico, California, and Connecticut have the longest survival times.

In Table 4, Hawaii was used for the referent group. Hawaii had a significantly increased risk of death compared to California (hazard ratio: 1.177; 95% confidence limits (1.066–1.300)); Connecticut (hazard ratio: 1.261; 95% confidence limits (1.143–1.390)); Georgia (hazard ratio: 1.257; 95% confidence limits (1.134–1.394)); Iowa (hazard ratio: 1.232; 95% confidence limits (1.119–1.356)); New Mexico (hazard ratio: 1.272; 95% confidence limits (1.151–1.406)).

This study's findings showed that New Mexico reported the longest mean survival time compared to Hawaii, which had the shortest mean survival time, demonstrating a five-year difference. This suggests that there are potential differences across states that affect survival time.

In 2010, SEER began collecting data on breast cancer receptor status. The breast cancer subtypes are defined by joint hormone receptor (HR; estrogen receptor [ER] and progesterone receptor [PR]) and human epidermal growth factor 2 (HER2) receptor status. “Not 2010+” reported cancer patients did not have such data collected. Between 1973 and 1980, American Indians/Alaska Natives experienced longer survival times for not 2010+ cancers at 221.49 months (SD = 164.73) compared to Blacks at 168.07 months (SD = 133.42) and Whites at 162.24 months (SD = 138.07). During 1981–1985, American Indians/Alaska Natives again had a longer survival time for not 2010+ cancers at 186.95 months (SD = 120.42) compared to Whites at 153.38 months (SD = 118.39) and Blacks at 104.21 months (SD = 111.17). Between 1986 and 1990, American Indians/Alaska Natives had a longer survival time for not 2010+ cancer at 171.94 (SD = 102.23) compared to Whites at 164.24 months (SD = 100.19) and Blacks at 140.09 (SD = 108.83). During 1991–1995, American Indians/Alaska Natives again experienced a higher survival time for not 2010+ at 160.44 months (SD = 79.58), compared to Whites at 149.81 months (SD = 78.89) and Blacks at 131.07 months (SD = 80.69). During 1996–2000, American Indians/Alaska Natives had a higher survival time for not 2010+ cancers at 128.60 months (SD = 49.83), compared to Whites at 121.55 months (SD = 54.77) and Blacks at 116.55 months (SD = 59.65). Between 2001 and 2005, each ethnic group reported similar survival rates. American Indians/Alaska Natives reported a slightly longer survival time for not 2010+ at 87.64 months (SD = 31.94) compared to Whites at 85.47 (SD = 32.17) and Blacks at 85.25 months (SD = 32.44). Between 2006 and 2011 more cancer ER statuses were reported, American Indians/Alaska Natives reported the highest survival time among not 2010+ cancers at 42.16 months (SD = 16.33) and lowest survival with HER2+/HR− at 9.10 months (SD = 7.70). Whites reported the highest survival time for not 2010+ breast cancer at 42.02 months (SD = 17.39) and the lowest survival for those diagnoses with HER2+/HR+ at 9.27 months (SD = 6.58). Among Blacks during the same period, the longest survival time was also among those diagnosed with not 2010+ cancers with 42.28 months (SD = 16.24), with the lowest survival time being for those diagnosed with triple negative cancers at 8.94 months (SD = 6.65). There was no stage of diagnosis reported between 1973–1985 and 2006–2011. However, between 1986 and 1990 the highest survival for Whites and American Indians/Alaska Natives was for stage I cancers. White patient survival time was 185.80 months (SD = 85.52) while 208.50 months (SD = 83.23) was the mean survival for American Indians/Alaska Natives. Blacks reported 173.33 months (SD = 92.43) for stage I but their highest survival was 257.00 months (SD = 4.24) at stage IIB. Stage IV cancers had the shortest survival times across all ethnic groups with 27.68 months (SD = 39.76) for Whites, 16.22 months (SD = 21.04) for Blacks, and 4 months for American Indians/Alaska Natives with only one patient reported. During 1991–1995, the highest survival reported for Whites and Blacks was stage I cancers. Whites reported 170.17 months (SD = 68.06) and Blacks reported 155.29 months (SD = 69.62). However, the American Indians/Alaska Natives reported highest survival for stage IIIA cancers at 205.67 months (SD = 20.30). Stage IV cancers had the shortest survival times at 32.32 months (SD = 49.54) for Whites, 53.25 months (SD = 92.89) for Blacks, and 55.33 months (SD = 90.60) for American Indians/Alaska Natives. For American Indians/Alaska Natives the highest survival time was among stage IIIA cancers at 205.67 months (SD = 20.30). Between 1996 and 2000, the highest survival time was among stage I cancers; for Whites the survival was 134.74 months (SD = 45.71), 141.77 months (SD = 43.68) for Blacks, and 138.95 months (SD = 41.00) for American Indians/Alaska Natives. The shortest survival during that period was stage IV cancers; American Indians/Alaska Natives reported a mean of 43.11 months (SD = 38.05), 35.64 months (SD = 47.65) for Whites, and 17.57 months (SD = 16.88) for Blacks. Between 2001 and 2005, the highest survival time among Whites and American Indians/Alaska Natives was in stage I cancer. Whites reported a mean survival time of 102.04 months (SD = 27.82) and American Indians/Alaska Natives reported 105.47 months (SD = 24.42). Blacks reported the highest survival rates with stage IIIA cancers at 122.00 months (SD = 1.414). Stage IV cancers had the shortest survival times for all ethnic groups during that period, Whites reported 19 months with only one patient reported, Blacks reported 44.34 months (SD = 43.17), and American Indians/Alaska Natives reported 44.00 months (SD = 48.06).

## 4. Study Limitations

SEER has collected cancer data for over thirty years from cancer registries throughout the United States; it is nationally recognized and considered reliable source of information on incidence, mortality, and other related variables. Although use of SEER lends this study strength, it is limited in the information it can provide. SEER lacks insight into many variables, such as social and economic factors, that affect the survival time of breast cancer patients and may explain much of the disparities experience by more disadvantaged groups.

## 5. Conclusions

This study concludes that there are several factors accounting for breast cancer survival including state, local, and individual level factors. For example, the age of diagnosis was greatly different for those living in Iowa compared to Georgia. This presented a seven-year difference in disease presentation. It is imperative to understand what differences are found in breast cancer prevention between those two states. Additionally, there are other factors that may account for the differences in age of diagnosis. Even more concerning is the stark differences in disease prognosis.

At the state and governmental level, future studies may compare state policies, preventive breast cancer systems, and the current state of the health care systems and their effect on the outcome of women diagnosed with breast cancer. However, it may also be important for future studies to address other demographic variables including income, education level, and health insurance status. To better understand patient survival, primary factors affecting patient prognosis should be addressed. These factors can be current medical treatment and prevention regimen that affect breast cancer diagnosis and treatment, and more indirect factors will also affect survival. Additionally, further investigation should be considered at the community level to determine what specific factors prevent the receipt of preventive screening or access to adequate care. This will inform the design and troubleshooting of prevention and treatment efforts.

These study findings give credence to the importance of early detection and treatment in reducing breast cancer incidence nationwide. Given the patient's current location and reported conditions, our findings suggest that geographical and local characteristics affect the survival rates of breast cancer patients. More in-depth research can help highlight factors contributing to the disparities seen across states. State health policies, access to health care, breast cancer screening and awareness programs, and cultural norms may affect screening and preventive care, which will likely affect long-term patient survival. The mean age at diagnosis ranged from 58 to 64 years, underlining the importance for following the United States Preventive Services Task Force (USPSTF) recommendations for biennial screening mammography for women aged 50 to 74 years. Breast cancer takes approximately 2 to 5 years to develop; therefore, women should be screened regularly to identify and treat precancers as well as prevent the progression of breast cancer. The findings in this study reinforce the importance of early detection and treatment in reducing breast cancer. Increasing patient survival first begins with improving screening efforts among poor and underserved women who may be at highest risk. However, it is important to recognize that some women, such as those with a family history of the disease, are at risk and may experience earlier presentations of breast cancer and should be screened earlier than the standard guidelines suggest. Public health and health care prevention efforts should target the most disadvantaged communities to help eliminate breast cancer-related health equity. For example, Black women experience more aggressive forms of breast cancer and are often diagnosed at early ages; therefore, this group may require more attention in screening and education efforts. This study showed that stage IV cancer had the lowest survival times among all groups. The highest survival times for Whites and American Indians/Alaska Natives were for stage I cancers, and the highest survival times for Blacks varied in the 5-year stratification analysis. Furthermore, economically disadvantaged patients that lack health insurance often do not receive adequate or appropriate care for their diagnosis adversely affecting their prognosis. This may include improving the treatment options for these groups and improving screening efforts to ensure earlier disease detection. Additionally, improving outreach efforts for programs such as NBCCEDP will help to target many underserved communities. Furthermore, high survival estimates in New Mexico, California, and Connecticut indicate the need to gather information about regional and environmental characteristics affecting the survival rates of breast cancer patients. The combination of current knowledge and previous research regarding predictive modeling can be used to inform policy decisions and to plan allocation of future resources and interventions [18–21]. As it relates to cancer prevention and control there are several other factors that have been associated with breast cancer risk including lifestyle practices such as alcohol and tobacco use, obesity, and physical inactivity. Medical therapies such as high duration of hormone therapy to treat menopause have also been associated with increased breast cancer risk. Consistent biennial screening and genetic testing for those who are genetically predisposed to breast cancer have also been strongly linked to reducing breast cancer risk and should be encouraged. However, many at risk groups also face issues of alcohol and tobacco use, poor diet, obesity, and physical inactivity which increase their risk of cancer. Therefore, these lifestyle changes should be addressed in more disadvantaged groups to help prevent and control the incidence of cancer. Additionally, women in these groups often experience no or inadequate health insurance coverage that provides affordable screening options. Therefore, there is more need to provide affordable screening options in minority and socioeconomically disadvantaged communities as well as providing nutrition and fitness counseling services that encourage healthy lifestyle choices that are reasonable and practical for economically disadvantaged communities that face greater barriers and poorer breast cancer outcomes.

## Figures and Tables

**Table 1:** Age at diagnosis and survival time for female breast cancer patients (*n* = 2,000).

	Statistics	California	Connecticut	Georgia	Hawaii	Iowa	New Mexico
Age at diagnosis (years)	Mean	61.42	62.51	57.97	59.72	64.14	61.30
	SE	0.564	0.320	0.313	0.305	0.324	0.316
	Median	60	62	57	59	65	61
	Quartiles	50, 60, 71	51, 62, 74	48, 57, 68	49, 59, 70	53, 65, 76	51, 61, 72

Survival time (months)	Mean	181.12	165.18	155.13	119.01	152.76	189.09
	SE	17.882	16.489	15.739	5.394	14.115	20.414
	Median	96	87	84	91	88	83
	Quartiles	41, 96, 175	35, 87, 167	34, 84, 158	37, 91, 167	38, 88, 167	33, 83, 158

**Table 2:** Race, ethnicity, and marital status for female breast cancer patients (*n* = 2,000).

	CA	%	CT	%	GA	%	HI	%	IA	%	NM	%
Race												
White	1589	79.45	1876	93.8	1403	70.15	604	30.2	1977	98.85	1894	94.7
Black	161	8.05	105	5.25	560	28	17	0.85	18	0.9	17	0.85
American Indian or Alaskan Native	4	0.2	1	0.05	3	0.15	4	0.2	0	0	75	3.75
Asian or Pacific Islander	230	11.5	8	0.4	31	1.55	1366	68.3	2	0.1	8	0.4
Other	8	0.4	4	0.2	0	0	4	0.2	0	0	2	0.1
Unknown	8	0.4	6	0.3	3	0.15	5	0.25	3	0.15	4	0.2
Ethnicity												
Hispanic	140	7	72	3.6	54	2.7	78	3.9	8	0.4	533	26.65
Non-Hispanic	1860	93	1928	96.4	1946	97.3	1922	96.1	1992	99.6	1467	73.35
Marital status												
Single	293	14.65	233	11.65	252	12.6	219	10.95	145	7.25	199	9.95
Married	1062	53.10	1059	52.95	1095	54.75	1192	59.6	1165	58.25	1082	54.1
Separated	25	1.25	85	4.25	19	0.95	15	0.75	8	0.4	10	0.5
Divorced	193	9.65	173	8.65	235	11.75	186	9.3	138	6.9	159	7.95
Widowed	370	18.50	375	18.75	342	17.1	328	16.4	512	25.6	364	18.2
Unknown	57	2.85	75	3.75	57	2.85	60	3	32	1.6	186	9.3

**Table 3:** Hazard ratio and confidence intervals.

States	DF	Parameter estimate	Standard error	Chi-Square	Pr > ChiSq	Hazard ratio	95% hazard ratio Confidence limits	
							Lower limit	Upper limit
California	1	−0.078	0.048	2.622	*p* = 0.105	0.925	0.842	1.017
Connecticut	1	−0.009	0.047	0.039	*p* = 0.844	0.991	0.903	1.087
Georgia	1	−0.012	0.050	0.057	*p* = 0.811	0.988	0.895	1.091
Hawaii	1	−0.241	0.051	22.318	*p* < 0.0001	0.786	0.711	0.869
Iowa	1	−0.033	0.046	0.490	*p* = 0.484	0.968	0.884	1.060

**Table 4:** Hazard ratio and confidence intervals.

States	DF	Parameter estimate	Standard error	Chi-Square	Pr > ChiSq	Hazard ratio	95% hazard ratio Confidence limits	
							Lower limit	Upper limit
California	1	0.163	0.051	10.400	*p* < 0.001	1.177	1.066	1.300
Connecticut	1	0.232	0.050	21.645	*p* < 0.0001	1.261	1.143	1.390
Georgia	1	0.229	0.053	18.834	*p* < 0.0001	1.257	1.134	1.394
Iowa	1	0.208	0.049	17.990	*p* < 0.0001	1.232	1.119	1.356
New Mexico	1	0.241	0.051	22.318	*p* < 0.0001	1.272	1.151	1.406

## References

[1] DeSantis C., Ma J., Bryan L., Jemal A. (2014). Breast cancer statistics. *CA: A Cancer Journal for Clinicians*.

[2] Siegel R., Ma J., Zou Z., Jemal A. (2014). Cancer statistics, 2014. *CA: A Cancer Journal for Clinicians*.

[3] Althuis M. D., Dozier J. M., Anderson W. F., Devesa S. S., Brinton L. a. (2005). Global trends in breast cancer incidence and mortality 1973–1997. *International Journal of Epidemiology*.

[4] Siegel R., Desantis C., Virgo K. (2012). Cancer treatment and survivorship statistics, 2012. *CA Cancer Journal for Clinicians*.

[5] ACS Cancer prevention and early detection: 10 key facts for 2015. http://www.cancer.org/research/acsresearchupdates/cancerprevention/cancer-prevention-and-early-detection-10-key-facts-for-2015.

[6] Dunn B. K., Agurs-Collins T., Browne D., Lubet R., Johnson K. a. (2010). Health disparities in breast cancer: biology meets socioeconomic status. *Breast Cancer Research and Treatment*.

[7] Howard D. H., Tangka F. K. L., Royalty J. (2015). Breast cancer screening of underserved women in the USA: results from the national breast and cervical cancer early detection program, 1998–2012. *Cancer Causes and Control*.

[8] Ward E., Jemal A., Cokkinides V. (2004). Cancer disparities by race/ethnicity and socioeconomic status. *Cancer Disparities*.

[9] Gross C. P., Long J. B., Ross J. S. (2013). The cost of breast cancer screening in the medicare population. *JAMA Internal Medicine*.

[10] Sanderson M., Levine R. S., Fadden M. K. (2015). Mammography screening among the elderly: A research challenge. *American Journal of Medicine*.

[11] Miranda P. Y., Tarraf W., González H. M. (2011). Breast cancer screening and ethnicity in the United States: implications for health disparities research. *Breast Cancer Research and Treatment*.

[12] Pruitt S. L., Lee S. J. C., Tiro J. A., Xuan L., Ruiz J. M., Inrig S. Residential racial segregation and mortality among black, white, and Hispanic urban breast cancer patients in Texas, 1995 to 2009. *Cancer*.

[13] Tian N., Wilson J. G., Zhan F. B. (2011). Spatial association of racial/ethnic disparities between late-stage diagnosis and mortality for female breast cancer: where to intervene?. *International Journal of Health Geographics*.

[14] Wheeler S. B., Reeder-Hayes K. E., Carey L. a. (2013). Disparities in breast cancer treatment and outcomes: biological, social, and health system determinants and opportunities for research. *The Oncologist*.

[15] Clarke C. A., Keegan T. H. M., Yang J. (2012). Age-specific incidence of breast cancer subtypes: understanding the black-white crossover. *Journal of the National Cancer Institute*.

[16] CDC United States Cancer Statistics: 1999–2011 Incidence and Mortality Web based reports. http://www.cdc.gov/cancer/breast/statistics/state.htm.

[17] SEER Surveillance, Epidemiology, and End Results (SEER) Program, Research data (1973–2011). https://seer.cancer.gov/.

[18] Khan H. M. R., Saxena A., Gabbidon K., Rana S., Ahmed N. U. (2014). Model-based survival estimates of female breast cancer data. *Asian Pacific Journal of Cancer Prevention?: APJCP*.

[19] Khan H. M. R., Saxena A., Gabbidon K., Ross E., Shrestha A. (2014). Statistical applications for the prediction of white hispanic breast cancer survival. *Asian Pac J Cancer Prev*.

[20] Khan H. M. R., Saxena A., Gabbidon K., Stewart T. S.-J., Bhatt C. (2014). Survival analysis for white non-Hispanic female breast cancer patients. *Asian Pacific Journal of Cancer Prevention?: APJCP*.

[21] Khan H. M. R., Saxena A., Vera V., Abdool-Ghany F., Gabbidon K., Perea N. (2014). Black hispanic and black non-hispanic breast cancer survival data analysis with half-normal model application. *Asian Pac J Cancer Prev*.

